# Plasmonic photothermal catalysis for solar-to-fuel conversion: current status and prospects

**DOI:** 10.1039/d1sc00064k

**Published:** 2021-03-12

**Authors:** Shunqin Luo, Xiaohui Ren, Huiwen Lin, Hui Song, Jinhua Ye

**Affiliations:** International Center for Materials Nanoarchitectonics (WPI-MANA), National Institute for Materials Science (NIMS) 1-1 Namiki Tsukuba Ibaraki 305-0044 Japan song.hui@nims.go.jp Jinhua.YE@nims.go.jp; Graduate School of Chemical Sciences and Engineering, Hokkaido University Sapporo 060-0814 Japan; Jiangsu Key Laboratory of Materials and Technology for Energy Conversion, Nanjing University of Aeronautics and Astronautics Nanjing 210016 P. R. China; TJU-NIMS International Collaboration Laboratory, School of Material Science and Engineering, Tianjin University Tianjin 300072 P. R. China

## Abstract

Solar-to-fuel conversion through photocatalytic processes is regarded as promising technology with the potential to reduce reliance on dwindling reserves of fossil fuels and to support the sustainable development of our society. However, conventional semiconductor-based photocatalytic systems suffer from unsatisfactory reaction efficiencies due to limited light harvesting abilities. Recent pioneering work from several groups, including ours, has demonstrated that visible and infrared light can be utilized by plasmonic catalysts not only to induce local heating but also to generate energetic hot carriers for initiating surface catalytic reactions and/or modulating the reaction pathways, resulting in synergistically promoted solar-to-fuel conversion efficiencies. In this perspective, we focus primarily on plasmon-mediated catalysis for thermodynamically uphill reactions converting CO_2_ and/or H_2_O into value-added products. We first introduce two types of mechanism and their applications by which reactions on plasmonic nanostructures can be initiated: either by photo-induced hot carriers (plasmonic photocatalysis) or by light-excited phonons (photothermal catalysis). Then, we emphasize examples where the hot carriers and phonon modes act in concert to contribute to the reaction (plasmonic photothermal catalysis), with special attention given to the design concepts and reaction mechanisms of the catalysts. We discuss challenges and future opportunities relating to plasmonic photothermal processes, aiming to promote an understanding of underlying mechanisms and provide guidelines for the rational design and construction of plasmonic catalysts for highly efficient solar-to-fuel conversion.

## Abstract 2

Hot carrier activation and photothermal heat can be constructively coupled using plasmonic photothermal catalysts for synergistically promoted solar-to-fuel conversion efficiency.

## Graphical abstract

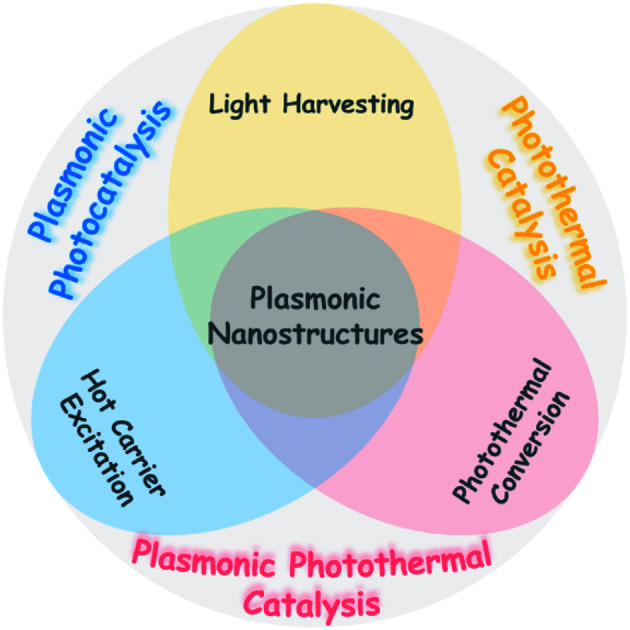

## 1. Introduction

Solar-energy is the most abundant renewable energy resource, and is one of the most promising candidates in the future renewable energy supply framework.^1,2^ Using solar-energy with photocatalysts to initiate catalytic reactions, the so-called photocatalytic process, is an emerging trend and regarded as a promising strategy to curtail increasing global energy demand while alleviating environmental issues.^3^ Adopting solar-energy solely as the energy input, CO_2_, H_2_O, and other building blocks can be transformed by photocatalysts into value-added chemicals, demonstrating the concept of solar-to-fuel conversion by efficiently storing the solar-energy in chemical bonds.^4,5^ Due to the wide band gap and limited light–matter interaction, conventional semiconductor-based photocatalysis faces two major challenges: limited photo harvesting and low apparent quantum efficiency (AQE). In our opinion, extending the range of optical response to improve solar light harvesting is highly desirable, and is considered to be an effective approach for enormously enhancing the net reaction efficiency. Previous work has suggested that plasmonic photocatalysis performs with attractive light absorption and charge carrier utilization features.^6,7^ In plasmonic photocatalysis, the free electrons in a plasmonic metal nanoparticle can be oscillated collectively under light irradiation through the excitation of localized surface plasmon resonance (LSPR).^8^ In addition to the electron–hole pairs generated from semiconductor photocatalysts, LSPR can serve as an additional energy input for enhanced photocatalytic performance by converting light energy into a hot carrier. Since the resonant wavelength and SPR intensity are determined by the size, shape, and composition of the plasmonic nanostructures, it is possible to fabricate plasmonic nanometals with full solar-spectrum response.^9,10^

Along with the plasmonic photocatalysts which combine plasmonic metals with semiconductors, utilizing plasmonic metals solely as catalysts has emerged as a cutting-edge research area of solar-to-fuel conversion technology.^11^ The unique capability of plasmonic metals that combines light-harvesting, hot carrier excitation, photo-to-thermal conversion, and surface catalytic reaction in one material endows them with a bright future for catalyzing various chemical reactions. With the assistance of both hot carriers and photothermal heat, several conventional catalytic processes which were originally driven by thermal-energy can be initiated by solar-energy *via* plasmonic photothermal technology without additional thermal energy input, even demonstrating superior activity compared with traditional thermocatalysis.^12–14^ Furthermore, the participation of a hot carrier in the surface reaction can preferentially activate desired chemical reactions through specifically targeting electronic excitations, offering great potential to discover additional and more selective reaction pathways that cannot be accessed by the conventional thermocatalytic process.^15^ These remarkable achievements strongly indicate that plasmonic photothermal technology that takes advantage of both the photon and the thermal features of sunlight holds great promise for addressing increasing global energy demands through solar-to-fuel conversion.

Previous reviews describing the reaction mechanism of plasmon-mediated photocatalysis and photothermal catalysis are already available, and those published reviews mainly included plasmonic or photothermal catalytic reactions for C_1_ molecules or conversion of other small molecules.^12,16–18^ Considering the advantages of plasmonic photothermal catalysis over other photocatalytic processes, an up-to-date perspective on current advances is necessary. In this perspective, recent advances in energetically uphill photosynthetic reactions (primarily including the transformation of chemical feedstocks CO_2_ and/or H_2_O into valuable fuels) driven by plasmonic photocatalysis, photothermal catalysis, and plasmonic photothermal catalysis are summarized. First, the concepts and reaction mechanisms of plasmonic photocatalysis, purely photothermal catalysis and plasmonic photothermal catalysis, including hot carrier transfer and photothermal conversion, are briefly introduced. Then, we comprehensively discuss the material design strategies and reaction mechanisms based on different plasmon-mediated endothermic reaction concepts: (i) hot carrier-mediated reaction at ambient temperature where the photo-induced hot carriers directly participate in the reaction without the contribution of photothermal heat (Section 3.1); and (ii) photothermal effect dominated catalytic reaction where most of hot carriers dissipate their energy into heat instead of activating the adsorbates (Section 3.2). Moreover, by elaborately constructing plasmonic photothermal catalysts, dual-functional roles (hot carrier excitation and photothermal heat) can synergistically contribute to the chemical reaction, and we particularly emphasize the important role of plasmonic photothermal catalysis in solar-to-fuel conversion (Section 3.3). The design of catalysts and approaches for improving energy conversion efficiency in this field are discussed. Finally, we conclude with the challenges and opportunities in future research on the current topic. This perspective aims to deliver conceptually novel and insightful views toward highly efficient solar-energy-driven catalytic chemical reactions.

## 2. Fundamentals of plasmonic photothermal catalysis

Surface plasmon resonance (SPR) is a common optical property of several metallic nanoparticles, which can be visualized as the coherent oscillation of conduction electrons established when the frequency of the photons matches the natural frequency of the surface electrons oscillating against the restoring force of positive nuclei.^15^ After the intra-band (between the Fermi level and the sp conduction band) or inter-band (between the d band and sp conduction band) excitation of plasmonic nanometals, energetic hot carriers are produced from the dephasing of the free electron oscillation through a non-radiative energy dissipation pathway (also known as the Landau damping process).^10^ Those hot carriers with sufficient energy can inject into the electron-accepting states of nearby surface adsorbates or semiconductors, inducing a surface chemical transformation (at femtosecond to picosecond level), or dissipate their energy into heat through the energy exchange between hot carriers and phonon modes (at picosecond to nanosecond level) to promote the mass transfer and reaction rate.^8,11^ Depending on the type of main driving force, the SPR-mediated catalytic process over plasmonic nanostructures can be divided into plasmonic photocatalysis and photothermal catalysis, where the former is dominated by charge carrier activation and the latter is governed by photothermal heat. It is noteworthy that through tailoring the optical properties (such as the energy of LSPR) of the plasmonic metal to match the electronic structure of the adsorbates, hot carrier activation and photothermal heat can be constructively coupled by the plasmonic photothermal catalysts under the irradiation of high-intensity light, ultimately initiating the surface reaction efficiently and selectively. Such a kind of reaction process is defined here as plasmonic photothermal catalysis that combines the advantages of plasmonic photocatalysis and photothermal catalysis. The reaction mechanism of plasmonic photothermal catalysis, including hot carrier transfer and photothermal conversion, is introduced briefly in the following section.

### 2.1 Reaction mechanism of plasmonic photocatalysis

**Fig. 1:**
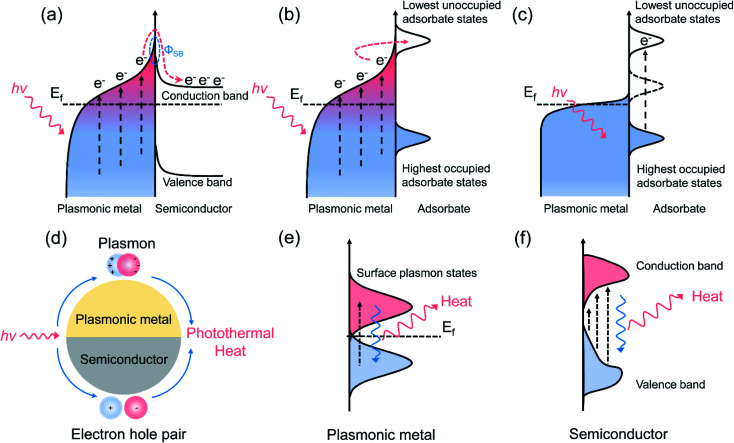
Schematic illustrations of the hot carrier transfer pathway and photothermal conversion. (a) A hot electron transfer process at a plasmonic metal/semiconductor interface. (b) The indirect hot carrier transfer mechanism. (c) The direct hot carrier transfer mechanism. (d) The photo-induced thermal effect of a plasmonic metal and semiconductor *via* plasmon- and exciton-based processes. (e) The photothermal conversion process over a plasmonic metal. (f) The photothermal conversion process over a semiconductor with LSPR characteristics.

Purely plasmonic photocatalytic reactions are generally conducted at ambient temperature or low reaction temperature (less than 100 °C), where photo-induced hot carriers play a dominant role in the reaction, and the influence of photothermal heat is insignificant during the plasmonic photocatalysis. In the plasmonic photocatalytic process, photo-induced hot electrons can be produced over a plasmonic nanoparticle and transfer to an adjacent semiconductor or interact with the molecules adsorbed on the surface of nanoparticle.^19–21^ For the plasmonic metal/semiconductor system (Fig. 1a), abundant hot electrons with high potential energy are generated from plasmonic metals *via* SPR excitation, followed by the efficient transfer of hot electrons to the conduction band (CB) of the interfaced semiconductors.^8,22^ The Schottky barrier (*Φ*_SB_) formed at the semiconductor–metal interface prevents the flowback of the hot electrons, extending the lifetime of the hot electrons and accumulated electrons in the CB of the semiconductor, which is beneficial to various surface chemical reactions, such as H_2_O splitting and CO_2_ reduction.^23–25^ On the other hand, for the plasmonic metal/adsorbate system, photo-induced hot carriers are in a distribution of low-energy charge carriers in high concentration. Hot carriers with a suitable energy level can be injected into the adsorbates through accessible orbitals, which is called an indirect hot carrier transfer mechanism (Fig. 1b).^26^ In addition, the hot carriers can be directly transferred from metals to adsorbates through a hybridized surface electronic state formed at the interface for activating the reactants, without the formation of the excited electron distribution (Fig. 1c).^27–29^ With the assistance of hot carriers, both the bond activation and the intermediate conversion can be facilitated through transient electronic excitations after photoexcitation of plasmonic nanostructures. In detail, hot carriers help the adsorbate molecules to evolve on the surface of plasmonic metal after energy transfer from plasmon to adsorbates along the excited potential energy surface, providing additional vibration energy to the adsorbates, and thus facilitating the chemical bond activation.^29^ The LSPR can be established when the light wavelengths are longer than the size of the plasmonic nanoparticles, and the generation process of photo-induced hot carriers has been described by theoretical calculations.^30^ By using a free electron mode, Nordlander and co-authors simulated the energy distribution of hot electrons and holes in an Ag nanoparticle, and the results indicated that a higher production rate and lower energy of hot carriers could be achieved over a larger nanoparticle.^31^ In general, with the increasing size of plasmonic nanoparticles, a red-shift of the LSPR absorption peak could be observed.^32^ The resonant wavelength and the LSPR intensity are dependent on both the intrinsic nature of plasmonic metals and the geometry of plasmonic nanostructures.^9,10^ Plasmonic nanostructures with sharp features, such as the corners of nanocubes and tips of nanocones, demonstrate significantly higher local electromagnetic field enhancements compared with spherical nanoparticles of similar size.^33^ Due to the lightning rod effect and reduced radiative damping, sharp features of plasmonic nanostructures can concentrate the incident light energy and increase the production of photo-induced hot carriers, which play important roles for activating surface chemical transformations.^34^ In additions, through changing the size and shape of plasmonic nanostructures, a shift in the electric field density could be induced, changing the oscillation frequency of the electrons, thereby inducing different cross-sections of the LSPR properties. Therefore, modulating the composition and geometry of the plasmonic nanostructures could ultimately result in an enhanced catalytic property.^35^ Among all the metallic elements, coinage elements (Cu, Ag, and Au) in group IB demonstrate the best SPR effect. The energy thresholds for the inter-band excitation of Cu and Au nanometals are 2.1 and 2.2 eV, respectively, indicating the visible light absorption capacity of Cu and Au.^36–38^

### 2.2 Reaction mechanism of pure photothermal catalysis

Solar-energy can also be converted into heat through photothermal conversion over plasmonic materials, providing sufficient thermal energy to stimulate the catalytic reactions (Fig. 1d).^39^ For plasmonic metallic nanostructures, upon light irradiation, the oscillation of conduction electrons dephases and produces hot carriers. An athermal charge-carrier distribution then is formed.^40,41^ Afterwards, hot carriers cool down through transferring energy to lattice phonons. That is, those energetic charge carriers which do not participate in the charge migration process of the surface reaction would finally dissipate their energy to the phonon modes, inducing a considerable temperature increase on the surface of the plasmonic nanometals (Fig. 1e).^15,42^ During the photothermal conversion process, the heat can be dissipated through particle–media interfaces after electron–phonon interaction. Therefore, the photothermal conversion efficiency is dependent on the medium and the particle size of the plasmonic nanostructures. Generally, reducing the particle size of plasmonic nanometals can lead to an enhancement in the photothermal conversion efficiency.^43^ For semiconductor nanomaterials with LSPR characteristics, the photo-induced electrons fall back to the lower energy states through releasing energy into photons or phonons, where the latter can interact with the lattice to produce heat (Fig. 1f).^44^ Assuming that most of the carriers' energy is converted into heat through energy exchange between electrons and phonons, the reaction obeys the same mechanism as conventional thermocatalytic reactions.^45^ Unlike plasmonic photocatalysis, pure photothermal catalytic processes generally occur in gas-phase reactions, and light with high intensity (orders of magnitude higher than solar flux) is required to produce high temperatures to initiate the chemical reactions. In pure photothermal catalysis, solar-energy is merely converted into thermal energy, and the activation of surface adsorbates by hot carrier is negligible.

### 2.3 Reaction mechanism of plasmonic photothermal catalysis

In plasmonic photothermal catalysis, through the rational design of plasmonic catalysts, hot carrier-driven reactant activation and photothermal heating co-exist and can be combined to synergistically drive the surface chemical reactions.^13,14,46^ As described above, initially formed hot carriers after light excitation could redistribute their energy through different plasmon decay pathways.^11^ Through electron–electron collisions, light-excited charge carriers over plasmonic metals could dissipate their energy, resulting in the secondary excitation of charge carriers; and this could lead to a Fermi–Dirac distribution of hot carriers near the Fermi level of plasmonic metals.^15^ High-energy charge carriers from such a distribution can participate in the surface chemical reactions through exchanging energy between plasmonic nanostructures and surface adsorbed reactants.^15^ Alternatively, adsorbate electronic states induced by strong interaction between plasmonic metals and adsorbates can serve as an additional channel for directly dissipating the LSPR energy.^29^ The prerequisites for realizing such hot electron activation processes have several aspects. First, accessible adsorbate orbitals must exist that could match with the electronic structure of plasmonic metals, or the presence of interfacial electronic states through the strong interaction between adsorbate and plasmonic metal that would enable an ultra-fast and momentum-reserved direct electronic excitation.^11^ In addition, an intense electric field at the surface of plasmonic metals after light irradiation, which can reroute the charge excitation process, is also considered a precondition for such an energy exchange process.^11^ Consequently, hot carriers can facilitate the surface reaction through the following reaction roles: (1) activating specific chemical bonds of surface reactants to modify the reaction pathway, which can enhance the selectivity and suppress unintended side reactions;^47^ (2) accelerating the desorption of certain surface-adsorbed species, which can further promote the catalytic activity and prolong the stability by resisting the coking of metallic nanoparticles.^48^ The residual hot carriers that do not participate in the surface catalytic reaction could subsequently cool down by transferring their energy to phonon modes, resulting in heating of the plasmonic metal surface.^11^ Photo-induced heat can also serve as an effective driving force for stimulating the surface reaction through the following functions: (1) exciting the phonon modes of the plasmonic nanoparticle, which could couple with a reaction coordinate, evolving the surface-adsorbed reactants to products;^12^ (2) promoting the adsorption–desorption process of the surface catalytic process;^39^ (3) shifting the chemical equilibria toward a higher yield of end products. Compared with plasmonic catalysis and photothermal catalysis, plasmonic photothermal catalysis is much more attractive since both the light and thermal features of solar light are concurrently utilized, resulting in highly efficient solar-to-energy conversion. Under such circumstances, the dual-functional roles of the plasmonic nanostructures (hot carrier-driven reactant activation and photothermal conversion) should be systematically considered and carefully differentiated.

There are several distinct signatures that can experimentally differentiate the contribution of hot carriers from photothermal heat: (1) a linear dependence of the reaction rate on light intensity indicates that the surface reaction is induced by hot carriers, and this can be attributed to the fact that the generation rate of a hot carrier increases linearly with the photon flux;^15^ (2) a higher kinetic isotope effect compared with the reaction driven by thermal energy at same reaction temperature;^49^ (3) modified selectivity for the chemical reaction induced by photo energy compared with the same reaction induced by thermal energy;^48^ (4) similar tendencies of apparent quantum efficiency and the optical absorption spectrum of plasmonic metal.^50^ The contribution of photo-induced hot carriers in the situation where the hot carriers and photothermal heat work synergistically can be evaluated through precisely designed contrast experiments.^14^ More specifically, through comparing the catalytic activity under purely solar-driven conditions (without additional thermal energy input) and under thermocatalytic condition (without irradiation of light) with external heating temperature equivalent to those achieved under illumination, the contribution of hot carriers can be differentiated quantitively.^14^ It should be noted that the surface temperature of the catalyst needs to be detected accurately in such a process.^51^

### 2.4 Solar-to-energy conversion efficiency

In order to evaluate the efficiency of plasmonic photothermal catalytic systems, two kinds of efficiency, *i.e.*, solar-to-fuel efficiency (STF) and apparent quantum efficiency (AQE), are introduced. For a thermodynamically uphill reaction, STF is an ideal method to calculate the conversion and storage efficiency of the solar light, whereas the AQE is suitable for both thermodynamically uphill and downhill reactions since it involves only the number of reacted electrons.

STF can be calculated using eqn (1).1
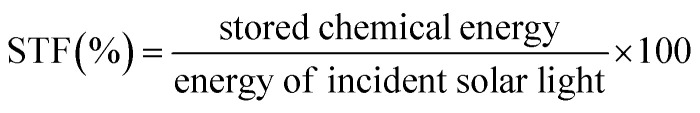


AQE can be calculated by using eqn (2).2
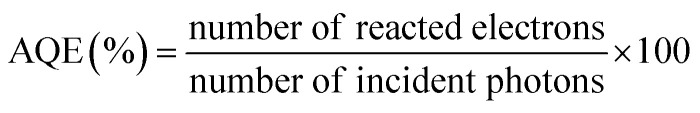


## 3. Solar-driven uphill reactions on plasmonic catalysts

### 3.1 Plasmonic photocatalysis

Metal nanoparticles with strong light scattering ability can generate hot electrons under ultraviolet, visible, and near-infrared light irradiation through the decay of surface plasmons. By applying hot electrons as the driving force to initiate the activation of adsorbates or intermediates, the performance of photocatalytic reactions can be greatly enhanced through two different mechanisms, as previously proposed: (1) enhanced light scattering with the presence of metals can improve the light trapping and absorption of semiconductors. Increasing the electron density in the metal can lead to near-field electric enhancement with increased interfacial charge transfer capacity; (2) optically excited hot electrons with sufficient energy can be injected into the conduction band of the semiconductor by overcoming the Schottky barrier and can take part in the reaction.^52^ With the assistance of hot carriers, various thermodynamically unfavorable reactions can be initiated effectively even at ambient temperature. In this section, we focus mainly on chemical reactions driven by photo-induced hot carriers, primarily plasmonic overall H_2_O splitting and CO_2_ reduction with H_2_O without the participation of photo-induced thermal energy.

#### 3.1.1 H_2_ production from water

**Fig. 2:**
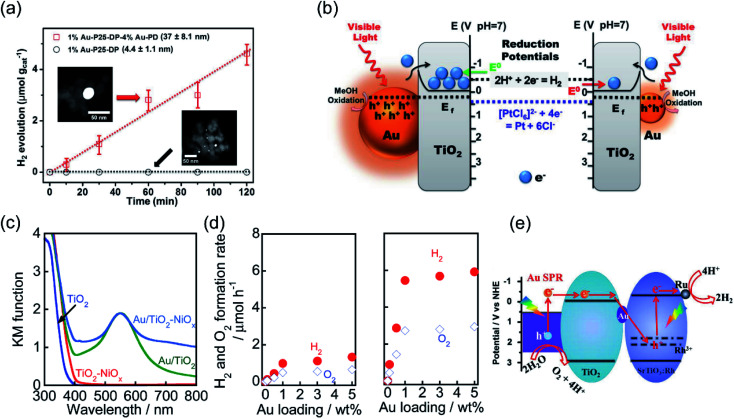
Plasmonic photocatalysis for overall water splitting. (a) The H_2_ evolution performance over Au–TiO_2_ with small-size and large-size Au under *λ* > 435 nm irradiation. (b) The proposed mechanism for the plasmon-mediated electron transfer process. (a and b) Reproduced from ref. 21 with permission from the American Chemical Society, Copyright 2014. (c) Light absorption spectra of TiO_2_, TiO_2_–NiO_*x*_, Au/TiO_2_, and Au/TiO_2_–NiO_*x*_ photocatalysts. (d) A comparison of Au–TiO_2_ and Ni-modified Au–TiO_2_ with different Au loading amounts for overall water splitting. (c and d) Reproduced from ref. 56 with permission from the Royal Society of Chemistry, Copyright 2017. (e) The proposed Z-scheme electron transfer model for pure water splitting. (e) Reproduced from ref. 57 with permission from Elsevier, Copyright 2017.

Water splitting driven by solar-energy is a fundamental step for artificial photosynthesis toward a sustainable energy future. Combining an oxide semiconductor with plasmonic metal nanostructures for water splitting is prevalent because the utilization of the hot carriers generated from metals can reduce the recombination of charge-carriers during charge-carrier migration. However, most studies of plasmonic photocatalysis have focused on the half reaction of water splitting, and organic molecules (such as methanol, ethanol, and lactic acid) are utilized as sacrificial agents during the reaction.^53,54^ In this perspective, we focus mainly on thermodynamically uphill reactions; therefore, hot carrier-assisted H_2_ production from pure water (without a sacrificial agent) is introduced in this section. In 2011, Chen *et al.* fabricated plasmonic Au–TiO_2_ as a photocatalyst, and investigated the reaction mechanism for SPR-mediated photocatalytic pure water splitting.^55^ After UV irradiation for 7 h, a small amount of H_2_ of about 0.79 μmol g^−1^ was produced from TiO_2_, while ∼44-fold enhancement (35.04 μmol g^−1^) has been achieved after incorporation of Au. Whereas, no H_2_ production was detected over Au–TiO_2_ under the visible light irradiation, probably because the SPR phenomenon of small Au nanoparticles (approximately 3 nm) is not significant. To clarify the important role of the size-dependent SPR effect of metal nanoparticles in photocatalysis, Qian *et al.* carried out comprehensive research on the water reduction performance of Au–TiO_2_ with different sizes of Au.^21^ Visible light (*λ* > 400 nm and *λ* > 435 nm) that is near the absorption tail of TiO_2_ was used as the light source. Under *λ* > 400 nm excitation, the amount of H_2_ generated over Au–TiO_2_ with small-size Au was 20 times higher than that over Au–TiO_2_ with large-size Au. However, under *λ* > 435 nm excitation, only Au–TiO_2_ with large-size Au produced a significant amount of H_2_ owing to the SPR effect of Au (Fig. 2a). Since no H_2_ production was detected over Au–TiO_2_ with small-size Au under *λ* > 435 nm irradiation, the authors suggested that for Au–TiO_2_ with small-size Au, the excited electrons were more likely to transfer from TiO_2_ and accumulate on Au active sites to retard the recombination of electron–hole pairs, as proposed in Fig. 2b. Meanwhile, increasing the nanoparticle size could significantly increase the light scattering, thus leading to the production of hot electrons for plasmon-mediated electron transfer and forming an extra electromagnetic field for enhancing activity. This work revealed that the efficiency of a plasmon-mediated electron transfer process was strongly governed by the size of the plasmonic metals, which could ultimately affect the reduction potential of the hot carriers. By varying the size of the plasmonic metals, the photocatalytic reaction could be facilitated efficiently. Furthermore, Ni-modified Au–TiO_2_ (with an average diameter of 13 nm) with strong Au SPR absorption at around 550 nm (Fig. 2c) was reported to show 5-fold enhancement (Fig. 2d) on overall water splitting under visible light irradiation, because the backward reaction between the evolved H_2_ and O_2_ into H_2_O could be suppressed by using NiO_*x*_.^56^ In this case, the apparent quantum efficiency (AQE) was calculated to be approximately 0.013%.

Further constructing a Z-scheme system based on Au–TiO_2_ has been proposed by Wang *et al.*^57^ Stable H_2_ (5.6 μmol h^−1^ g^−1^) and O_2_ (2.7 μmol h^−1^ g^−1^) evolution was achieved on Au–TiO_2_/Rh–SrTiO_3_ under visible light irradiation. Benefiting from electron accumulation, the plasmon-induced hot electrons generated in Au can transfer through the Z-scheme electron pathway and recombine with the holes in SrTiO_3_, as illustrated in Fig. 2e, which may lead to a decrease in surface potential and efficient charge separation for achieving overall water splitting. By controlling the length and width (aspect ratio) of Au nanorods, the SPR absorption range can be extended from the visible (600 nm) to the longer wavelength NIR (1200 nm) region. Elbanna *et al.* prepared Au nanorods with different aspect ratios (2.3, 3.2, 4.8 and 7.5) through a seed-growth method.^58^ After loading Au nanorods on TiO_2_ mesocrystals, the longitudinal SPR absorption peak red shifted from 660 to 975 nm, resulting in higher H_2_ production rate (924 μmol h^−1^ g^−1^) and prolonged electron lifetime under Vis-NIR light irradiation.

**Fig. 3:**
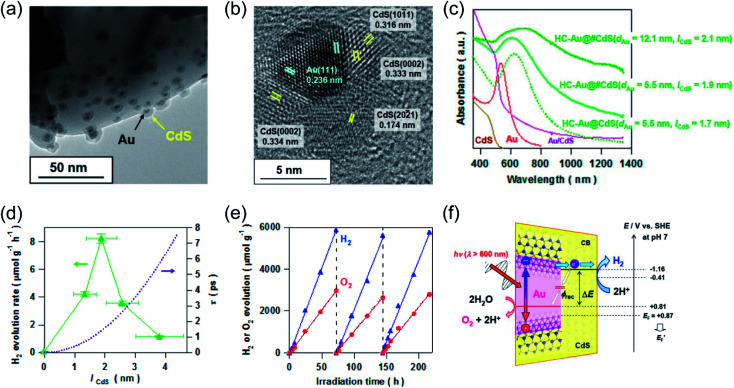
Plasmonic photocatalysis for overall water splitting. (a) and (b) TEM images of a ZnO supported Au@CdS heteroepitaxial junctional structure. (c) UV-vis-NIR absorption spectra of Au@CdS. (d) H_2_ evolution rate and electron transit time values in relation to CdS thickness. (e) Stability test over the optimal Au@CdS sample under red-light illumination. (f) The proposed LSPR-induced hot electron transfer mechanism. (a–f) Reproduced from ref. 59 with permission from the American Chemical Society, Copyright 2018.

Recent studies have shown that the LSPR excitation of Au–CdS hybrids also assisted electron transfer between the interface of Au and CdS. Naya *et al.* prepared selective deposited hexagonal CdS (shell) above Au nanoparticles (core) to form an Au–CdS heteroepitaxial junctional structure (Fig. 3a and b) with different mean sizes (*d*_Au_) and shell thicknesses (*l*_CdS_).^59^ As shown in Fig. 3c, the Au LSPR showed strong damping around 620 nm in contrast to the original Au signal with the peak located at *λ* = 520 nm. The H_2_ production activity was controlled by both the lifetime (*τ*) of SPR-electrons as well as the size of the CdS nanoparticle (Fig. 3d). Au–CdS (*d*_Au_ = 12.1 nm, *l*_CdS_ = 2.1 nm) gave a continuous H_2_ generation rate of (79.2 ± 2.1 μmol h^−1^ g^−1^) with an external quantum yield of 0.24% under excitation by red-light (*λ* = 640 nm). Benefitting from SPR, hot electrons generated from Au under red-light irradiation can transfer to CdS, endowing a stable stoichiometric water splitting of H_2_ and O_2_ (with production ratio 2 : 1) for more than 200 h (Fig. 3e and f). The above study further verified that the geometry and the dimension of the plasmonic nanostructures determine the overall efficiency toward water splitting.

In addition to Au, Ag-modified TiO_2_ exhibited an absorption peak in the visible region, and the bandgap was reduced from 3.0 to 2.5 eV due to the SPR effect. H_2_ generation from pure water was dramatically enhanced over Ag-modified TiO_2_ (470 μmol h^−1^ g^−1^) in comparison to bare TiO_2_ (206 μmol h^−1^ g^−1^) under UV light. The co-existence of metallic Ag and Ti–Ag–O species can trigger the SPR effect and form a new electron pathway for efficient electron–hole pair separation.^60^

#### 3.1.2 CO_2_ reduction

Chemical reduction of CO_2_ into value-added products by photocatalysis is currently attracting much attention as a promising technology for next-generation energy supply. Early studies indicated that loading plasmonic metal nanostructures such as Au, Ag nanoparticles can enhance the performance of photocatalytic CO_2_ reduction with H_2_O or H_2_ into CO, CH_3_OH, and other hydrocarbons under visible light irradiation.^61–64^ Notably, bimetallic structures can facilitate efficient electron transfer between two metallic parts, leading to a prolonged hot carrier lifetime for highly efficient plasmonic photocatalysis. Tahir *et al.* prepared a plasmonic Au–Ag alloy as a cocatalyst for improving the charge separation of TiO_2_ and investigated selective photocatalytic CO_2_ reduction under visible light irradiation.^65^ Benefitting from the synergistic effect of broad-spectrum absorption and efficient electron–hole separation, the yield rate of CO over the Au–Ag loaded TiO_2_ (1813 μmol h^−1^ g^−1^) was higher than that over Au–TiO_2_ (1053 μmol h^−1^ g^−1^), Ag–TiO_2_ (983 μmol h^−1^ g^−1^) or bare TiO_2_ (43 μmol h^−1^ g^−1^). Moreover, the amount of CO produced under visible light was 3.28-fold higher compared to UV-light irradiation, while an appreciable amount of CH_3_OH was also detected under visible light irradiation. The authors proposed that the surface electrons on the plasmonic Au–Ag alloy can be excited to a higher energy level and transferred to the conduction band of TiO_2_. Meanwhile, photogenerated electron–hole separation can also be promoted by small-size Au/Ag, synergistically promoting the photocatalytic performance of Au/Ag–TiO_2_. Plasmonic Au nanoparticles as photocatalysts were also found to be active under green-light-excitation, and the C_1_–C_3_ hydrocarbons were synthesized from CO_2_ and H_2_O assisted by the ionic liquid.^66^ Upon light excitation, a charge-rich environment at the interface between the plasmonic metal and the solution can be induced, which could facilitate CO_2_ activation. In addition, the intermediates formed at the surface were stabilized by the ionic liquid, facilitating the production of C_2_ and C_3_ chemicals.

**Fig. 4:**
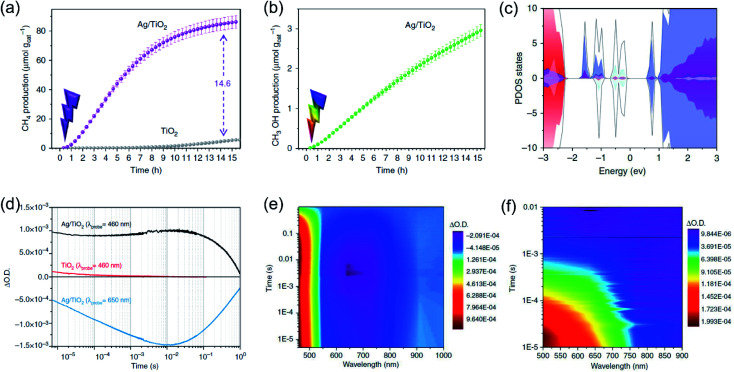
Plasmonic photocatalysis for CO_2_ reduction. (a) The cumulative production of CH_4_ under 365 nm irradiation. (b) The cumulative production of CH_3_OH under 450 nm irradiation. (c) Total DOS (black) and atom-projected DOS (PDOS) for Ag 5s (light blue), Ag 5d (grey), and Ti 3d (purple). (d) Temporal profiles of the transient absorption decay of Ag/TiO_2_ and TiO_2_ films after excitation at 355 nm. (e) A two-dimensional plot of the transient absorption spectrum of Ag/TiO_2_ film after UV excitation. (f) A two-dimensional plot of the transient absorption spectrum of Ag/TiO_2_ film excited with visible light. (a–f) Reproduced from ref. 67 with permission from Springer Nature, Copyright 2018.

Whilst the physical properties of LSPR effects are well understood, the reaction mechanisms of every process in the overall reaction over the real surfaces of catalysts are still controversial. In order to explain interfacial charge dynamics in photocatalytic reactions over plasmonic metal/semiconductor nanocomposites, a combination of *in situ* near ambient pressure XPS measurements and time-resolved optoelectronic characterization were conducted by O'Shea and co-authors.^67^ In their work, the reaction mechanism of photoreduction of CO_2_ using H_2_O as electron donor over plasmonic Ag/TiO_2_ nanostructures was systematically investigated. Under UV-light irradiation, an approximately 15-fold enhancement in the CH_4_ generation rate over Ag/TiO_2_ compared with bare TiO_2_ was found (Fig. 4a). In contrast, under visible light irradiation, the main product was shifted to CH_3_OH over Ag/TiO_2_ (Fig. 4b). Based on the operando spectroscopic measurements and theoretical calculations, the authors indicated that the surface sub-band gap states formed between the plasmonic Ag nanoparticles and TiO_2_ through interfacial dielectric coupling was an important factor to promote the separation of photo-induced charge carriers (Fig. 4c). The production of CH_4_ over Ag/TiO_2_ under UV light irradiation can be attributed to the electron-scavenging ability of Ag nanoparticles, resulting in an increased lifetime of photo-induced charge carriers compared to the original TiO_2_ (Fig. 4d and e). Under UV light irradiation, photo-induced electrons from TiO_2_ could be scavenged by Ag nanoparticles, while the holes could be accumulated at the valence band of TiO_2_, resulting in the spatial separation of photo-induced electron–hole pairs and an increased lifetime. The accumulation of photo-induced electrons on the surface of the Ag nanoparticle can facilitate the production of electron-demanding products (CH_4_) over Ag/TiO_2_. Furthermore, the longer lifetime of photo-induced electron–hole pairs enabled water to be the electron source for CO_2_ reduction (Fig. 4d). *In situ* near ambient pressure XPS measurements further verified the formation of C–H intermediates, indicating that the reaction pathway followed the carbene mechanism. Under visible light irradiation, photo-induced electronic transitions from intra-band gap surface states were induced, generating charge carriers in the near-surface region. Compared with the charge-carriers induced by UV light irradiation, those visible light-induced carriers showed shorter-lived transient absorption decay (Fig. 4f), shifting the reaction selectivity to lower electron-demanding products (CH_3_OH). This work provides an unequivocal explanation of the interfacial charge dynamics and a basic understanding of each process in the CO_2_ photoreduction.

Very recently, Sharma and co-authors reported that a strong endothermic reaction CO_2_ + C → 2CO which requires a high activation energy (3.1 eV) can be realized at ambient reaction temperature, and the LSPR energy of Al in the deep-UV range was used as the sole driving force to initiate the reaction without additional thermal energy input.^68^ The localized surface plasmon (LSP) mode of the Al nanoparticle was excited by an electron beam in an environmental scanning transmission electron microscope. The presence of LSP modes was characterized by using the electron energy loss spectroscopy (EELS) spectrum, and the spatial distribution was stimulated by using the discontinuous Galerkin time-domain (DGTD) method. The reaction product was detected by *in situ* gas chromatography-mass spectrometry (GC-MS). The reaction rate was estimated by measuring carbon gasification near the Al nanoparticle. Under electron beam excitation, an electric field can be induced on the side of the Al nanoparticle. After electron beam illumination, a new receded edge of the graphite flake was formed, which may prove the consumption of carbon during the reaction. From the GC-MS measurements, measurable CO signals were detected both after electron beam illumination at room temperature and after heating at a reaction temperature of 900 °C, confirming that the reaction did happen in both cases. The authors proposed that the plasmon of Al with high energy played a dominant role in driving the reaction at ambient temperature. In this work, a direct excitation and observation of electron-excited LSPR of a plasmonic Al nanoparticle were realized by using an environmental transmission electron microscope. In such a process, both the spatial distribution of LSP modes and the dynamic behavior of the plasmonic nanoparticles are characterized simultaneously. This work demonstrates a new path towards driving other industrially tough reactions at ambient temperature by using a plasmon.

Currently, it is of great interest to use noble metals as plasmonic materials not only for improving light absorption capacity but also for inducing hot carriers to promote catalytic performance over plasmonic photocatalysis. By modulating the species, size, and geometry of plasmonic nanostructures, enhanced photocatalytic activity can be observed. It should be noted that isotope measurements are necessary to confirm that the products indeed come from the reactants, especially when the amounts of the products are quite low.

### 3.2 Photothermal catalysis

Another strategy to convert solar-energy into chemical energy is *via* photo-thermochemistry, which utilizes the full spectrum of sunlight to increase the surface temperatures of catalysts and then initiate thermocatalytic reactions.^69^ Under these circumstances, photo-induced hot carriers play an insignificant role in activating the surface adsorbed reactants, but dissipate their energy into heat through energy exchange between electrons and phonons. Group VIII nanometals are considered promising candidates for photothermal catalysis because of their outstanding photothermal conversion capacity and catalytic property, although their SPR intensities are relatively weak.^70^ Due to the high photothermal conversion efficiency of plasmonic photothermal catalysts, the surface temperatures of catalysts can be elevated to 300–600 °C under irradiation by focused solar light, providing sufficient energy to stimulate thermodynamically unfavorable reactions, such as CO_2_ reforming with methane and reverse water gas shift.^71–73^

#### 3.2.1 CO_2_ reduction with H_2_

CO_2_ can be reduced with H_2_ to produce various chemicals, such as CH_4_ (CO_2_ methanation),^74,75^ CH_3_OH (methanol synthesis),^76,77^ and CO (reverse water gas shift)^78,79^ over photothermal catalysts. Aiming at demonstrating the concept of solar-energy conversion, reverse water gas shift (RWGS) with its endothermic nature is highlighted in this section. RWGS (CO_2_ + H_2_ → CO + H_2_O, Δ*H*_298 K_ = 41.2 kJ mol^−1^) is an attractive strategy for converting excessive CO_2_ in the atmosphere to CO, and then the CO can be utilized directly as feedstock for fuel manufacture (*e.g.* Fischer–Tropsch reaction).^80,81^ Supported metallic catalysts, such as noble metals (Pt, Pd), transition metals (Cu, Co), and oxide catalysts (ZnO/Al_2_O_3_, In_2_O_3_/CeO_2_), have been extensively investigated as promising candidates for thermocatalytic RWGS.^82–87^ In order to achieve a satisfactory CO_2_ conversion rate, a massive amount of non-renewable energy has to be indispensably invested in the thermocatalytic RWGS reaction. Compared with the strict conditions required by the thermocatalytic RWGS reaction, the utilization of photothermal technology has attracted tremendous research interest because of its milder reaction condition requirements.

Photothermal catalytic CO_2_ reduction over group VIII elements (such as Ru, Rh, Ni, Co) was systematically investigated previously by Ye and co-authors, and the reaction rates for photothermal CO_2_ reduction (mol h^−1^ g^−1^) were found to be several orders of magnitude larger than for conventional photocatalysis.^88^ The main products of CO_2_ reduction were CH_4_ and CO. A maximum CO_2_ reaction rate (18.16 mol h^−1^ g^−1^) was detected over metallic Ru, while Fe demonstrated the best selectivity towards photothermal catalytic RWGS. The authors indicated that the outstanding catalytic performance for VIII elements can be attributed to their excellent photothermal conversion capacity and outstanding catalytic activity. Pd@Nb_2_O_5_ was also found to exhibit good catalytic activity towards RWGS.^89^ After light irradiation, photothermal heat induced over Pd nanocrystals served as the driving force for stimulating RWGS, and a CO production rate of 1.8 mmol g^−1^ h^−1^ was observed.

**Fig. 5:**
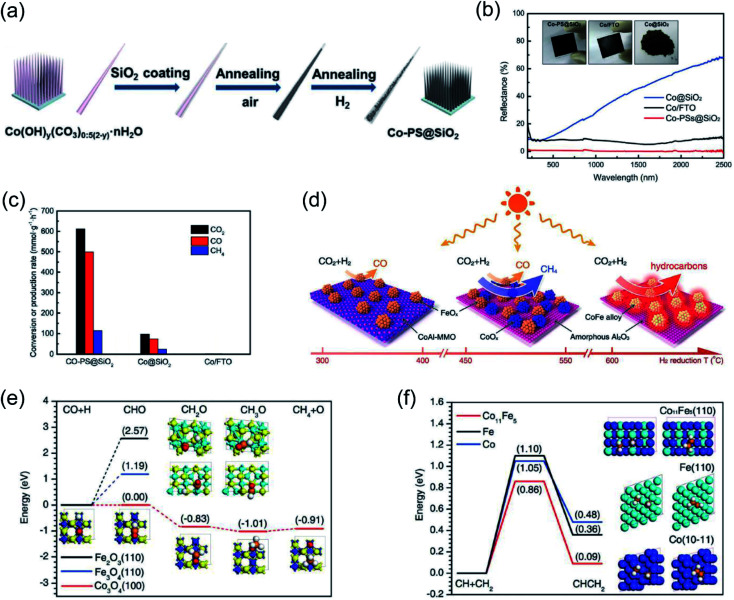
Photothermal catalytic CO_2_ reduction. (a) A schematic illustration of the fabrication process of Co-PS@SiO_2_. (b) The optical absorption properties of different cobalt catalysts. (c) The catalytic behavior of different samples normalized by the mass of Co. (a–c) Reproduced from ref. 90 with permission from John Wiley and Sons, Copyright 2020. (d) Different catalysts formed *via* H_2_ reduction at different temperatures and their selectivity toward solar-driven CO_2_ reduction. (e) The reaction paths for CO hydrogenation over different catalysts. (f) The probable C–C coupling path over different catalysts. (d–f) Reproduced from ref. 92 with permission from John Wiley and Sons, Copyright 2018.

Ozin and co-authors recently reported the fabrication of a nanoneedle-array-based cobalt plasmonic superstructure (Co-PS@SiO_2_) for photothermal catalytic CO_2_ reduction.^90^ In Co-PS@SiO_2_, each nanoneedle was composed of Co nanocrystals and encapsulated by silica (Fig. 5a), which enabled nearly 100% light absorption efficiency (Fig. 5b). Under intense light irradiation, Co nanocrystals were heated to high temperature through the photothermal effect. Compared with Co@SiO_2_, L-Co@SiO_2_, and Co/FTO, the as-prepared Co-PS@SiO_2_ obtained the highest surface temperature of 383 °C under 20 Suns illumination, indicating the outstanding photon harvesting and photothermal conversion capacity of Co-PS@SiO_2_. A photothermal CO_2_ hydrogenation reaction was conducted as the model reaction to evaluate the catalytic activity. The main products for photothermal CO_2_ hydrogenation over Co-based catalysts were CO and CH_4_, and the conversion rate of CO_2_ over Co-PS@SiO_2_ was measured to be 0.6 mol g^−1^ h^−1^ (normalized by the mass of Co) (Fig. 5c). Based on surface analysis and mechanistic studies, the authors indicated that a higher local temperature induced by intense light was essential for the observed higher activity of Co-PS@SiO_2_. This work provides an innovative strategy to promote photothermal conversion through constructing plasmonic nanostructures with antireflection effects, enabling a complete harvesting of the solar-energy towards highly efficient CO_2_ reduction.

It was also reported that the photon–matter interaction can enhance the photothermal conversion efficiency over plasmonic nanostructures, thus promoting the photothermal catalytic RWGS reaction. Ouyang and co-authors employed atomic-scale dispersed Co–N species anchored Co@C hybrid structure (Co@CoN&C) nanomaterials for CO_2_ photothermal reduction, and a CO_2_ conversion rate of 132 mmol g^−1^ h^−1^ with suppressed undesirable methanation was achieved in a batch-type reaction system under the irradiation of a 300 W Xe lamp.^91^ However, under the same conditions, Co nanoparticles demonstrated a reaction rate of only 27 mmol g^−1^ h^−1^. The temperature of the catalysts during the reaction was measured with an infrared thermometer, and a surface temperature of approximately 518 °C was detected over Co@CoN&C, higher than over Co nanoparticles (330 °C) under the same conditions. These results strongly indicated that Co@CoN&C exhibited higher photothermal conversion efficiency, which favored photothermal CO_2_ reduction. Contrast experiments suggested that the high selectivity could be attributed to the advantageous selectivity of the CoN&C shell, and the efficient photothermal conversion over the hybrid interfacial structure further promoted the catalytic activity. Based on chemisorption data and surface analysis results, the authors proposed that the enhanced CO_2_ adsorption capacity and the alleviated hydrogenation of CO_2_ into HCOO* synergistically facilitated the high conversion rate of CO_2_. This study further verifies that the thermodynamic and kinetic factors of a photochemical reaction can be modulated by the rational design of microstructures.

Through changing the reduction temperature in an H_2_ atmosphere, a series of CoFe-based catalysts can be derived from CoFeAl layered-double-hydroxide (LDH) materials, and they were applied in photothermal catalytic CO_2_ reduction. As presented by Zhang and co-authors, the chemical composition and morphology of CoFe catalysts can be modulated by changing the reduction temperature, which can ultimately control the reaction selectivity.^92^ With an increase in reduction temperature, the reaction products from photothermal catalytic CO_2_ reduction over CoFe catalysts shifted from CO to CH_4_, and eventually to C_2+_ products (Fig. 5d). A comparison of CO_2_ conversion rates over CoFe nanoparticles under photothermal heating and direct thermal heating without light irradiation at the same reaction temperature indicated that photothermal heat played a dominant role in the reaction. The authors proposed that the CoFe alloy nanoparticles are responsible for the high selectivity and photothermal effects. According to density functional theory (DFT) calculation results, the authors indicated that during the CO_2_ hydrogenation process, Fe-based catalysts favored the production of CO, whereas Co-based materials favored the production of CH_4_ (Fig. 5e). Furthermore, a C–C coupling reaction was promoted by CoFe bimetallic alloy, resulting in the production of C_2+_ products (Fig. 5f).

#### 3.2.2 CO_2_ reduction with H_2_O

In addition to CO_2_ reduction by H_2_ through the RWGS reaction, artificial photosynthesis using renewable solar-energy to reduce CO_2_ by H_2_O for fuel production (such as CH_4_, CH_3_OH, and other hydrocarbons), is considered to be the ultimate strategy for mitigating the greenhouse effect and solving energy shortages simultaneously.^12,93^ Several semiconductor photocatalysts have been investigated for solar-energy-driven CO_2_ reduction with H_2_O, such as C_3_N_4_/TiO_2_, and WO_3_.^94,95^ However, due to the strong endothermic nature of CO_2_ reduction by H_2_O, the photocatalytic activity for such a reaction is far from practical applications. Very recently, a few studies utilizing photothermal technology for driving artificial photosynthesis have been reported as promising strategies for enhancing the activity dramatically. For instance, Li and co-authors adopted a plasmonic MoO_3−*x*_ heterostructure and found that the light absorption range can be extended to the infrared region through the introduction of oxygen vacancies.^96^ MoO_3−*x*_ displayed good activity towards the photothermal catalytic conversion of CO_2_ into CO and CH_4_ without external sacrificial agents. After 4 h of full-spectrum irradiation, the yields of CO and CH_4_ over MoO_3−*x*_ reached 41.2 and 8.3 μmol g^−1^, which were 20 and 49 times higher than that of pristine MoO_3_. Based on contrast experiments, surface analysis, and theoretical calculation results, the authors proposed that the barrier for CO_2_ hydrogenation was decreased by oxygen vacancies in MoO_3−*x*_. In fact, CO_2_ reduction with H_2_O by using photothermal technology is still in its preliminary stages, and very few papers have reported reliable results when using H_2_O as a reducing agent to reduce CO_2_. Due to the low activities of products from CO_2_ reduction, isotope experiments are highly recommended to probe the carbon source in the products. Moreover, the reported net efficiencies are quite low, making them unfeasible for practical applications. Therefore, further efforts are required to explore a more efficient technology for CO_2_ reduction with H_2_O.

#### 3.2.3 CO_2_ reduction with CH_4_

CO_2_ reduction with CH_4_ (CO_2_ + CH_4_ → 2CO + 2H_2_ Δ*H*_298 K_ = 247.3 kJ mol^−1^), also known as dry reforming of methane (DRM), is a promising approach for converting two major greenhouse gases (CO_2_ and CH_4_) into syngas (CO and H_2_). Thermodynamically, DRM requires a very high temperature to overcome the activation energy barrier, consuming a large amount of energy. The catalysts are also vulnerable to deactivation in the harsh reaction conditions. Therefore, it is highly desirable to explore novel routes regarding energy input and catalyst design.

Recently, using a photothermal route for driving CO_2_ reduction with CH_4_ has attracted tremendous research interest. Group VIII metals (such as Ni, Co, and Pt) have been regarded as ideal catalysts for photothermal catalytic DRM.^73,97,98^ For example, Li and co-authors designed silica-cluster-modified Ni nanocrystals (SCM-Ni/SiO_2_) for highly efficient photothermal catalytic DRM.^97^ Under irradiation by focused light of high intensity, the surface temperature of SCM-Ni/SiO_2_ could reach approximately 650 °C from photothermal conversion. Photo-induced heating served as the driving force to initiate photothermal catalytic DRM without additional thermal energy input. Under full-spectrum light irradiation, high production rates of H_2_ and CO (17.1 and 19.9 mmol min^−1^ g^−1^) with a solar-to-fuel conversion efficiency up to 12.5% can be achieved. Density functional theory (DFT) calculation was conducted *via* the constrained occupancy approach to calculate the activation energy (*E*_a_) for each elementary step both in the excited state and the ground state during solar-driven DRM over an Ni/SiO_2_ slab, and the results revealed that the *E*_a_ for CH and CHO dissociation in the excited state were decreased (from 0.67 and 1.11 eV to 0.58 and 0.99 eV, respectively). In addition, the *E*_a_ for C and CH oxidation in the excited state also showed a slight decrease compared with the *E*_a_ in the ground state, suggesting the existence of a photoactivation effect which was responsible for the acceleration of DRM. In addition, SCM-Ni/SiO_2_ demonstrated excellent durability. After 700 h of reaction, no carbon deposition or deactivation could be observed over SCM-Ni/SiO_2_. Based on mechanistic investigations, the authors concluded that the highly efficient catalytic activity of SCM-Ni/SiO_2_ towards solar-light-driven DRM can be attributed to surface plasmon-induced photothermal conversion and an IR heating effect. The high surface temperature on the catalytically active Ni nanocrystals was responsible for the high activity.

In summary, CO_2_ conversion can be driven efficiently through photothermal technology without additional energy input. Various plasmonic nanostructured materials, such as Co-PS@SiO_2_, Co@CoN&C, or CoFe alloys, can be adopted as photothermal catalysts for CO_2_ conversion. By using photothermal technology, CO_2_ can be reduced by H_2_, H_2_O or CH_4_, and be converted into energy-containing fuel gas. Mechanistic investigations indicated that the full solar-spectrum, especially infrared light, can be absorbed to heat the catalysts to a high temperature through photothermal conversion, which promoted the activation of reactants and initiated thermodynamically unfavorable reactions such as RWGS and photosynthetic processes.

### 3.3 Plasmonic photothermal catalysis

After the oscillated electrons have been excited under light irradiation over plasmonic metals, the high energy electrons can relax to the ground state by a non-radiative decay route, following the Frank–Condon principle. The photo-induced hot carriers can either interact with molecules adsorbed on the surface of plasmonic metals by the selective activation of the chemical bonds of the adsorbates, or they can dissipate their energy into the environment to increase the local temperature. Recently, several studies have demonstrated that by the rational design of plasmonic photothermal catalysts, the hot carriers and photothermal heating can contribute to the reaction simultaneously, resulting not only in enhanced activity but also in modulated selectivity.^42,99,100^

#### 3.3.1 H_2_ production from water

A recent study has suggested that solar-energy could be considered as an efficient stimulus for water splitting, and both the charge carriers and photothermal heat contributed to the reaction in concert.^101^ In a typical study, SiO_2_/Ag@TiO_2_ core–shell nanocomposites were designed and applied as a solar thermal collector to produce clean H_2_ from glycerol aqueous solution.^101^ Contrast experiments using the illuminant in different wavelength ranges revealed that an H_2_ production rate of 786 μmol g^−1^ h^−1^ was achieved over SiO_2_/Ag@TiO_2_ under UV light irradiation, which was higher than for SiO_2_@TiO_2_ or TiO_2_ spheres. All the samples were detected at similar temperatures (approximately 30 °C) under irradiation by UV light, illustrating that the photothermal effect played a secondary role in this case. After the illuminant was changed to full-spectrum, the reaction rate of SiO_2_/Ag@TiO_2_ increased enormously to 1536 μmol g^−1^ h^−1^. The significant enhancement in the catalytic activity, especially for SiO_2_/Ag@TiO_2_, was attributed to the photothermal effect since a relatively large temperature difference of 2.6 °C between SiO_2_/Ag@TiO_2_ and SiO_2_@TiO_2_ was detected by an IR camera after full-spectrum irradiation. However, no H_2_ was detected for any sample when visible-NIR light was solely used for irradiation. The authors proposed that the UV portion of the solar spectrum could be absorbed by the TiO_2_ porous shell, while the SiO_2_/Ag core absorbed visible and NIR light. In such a photocatalytic system, higher energy photons were used for electron–hole pair generation, while lower-energy photons were used to heat the catalysts and enhance the hole-scavenging effect of glycerol by the photothermal effect, achieving the full-spectrum utilization of solar light for photocatalytic H_2_ production. In addition, it is noteworthy that the actual surface temperatures of metallic nanoparticles in the liquid solutions might be higher than that of the liquid solutions detected by the IR camera. Although some impressive progress on plasmonic photothermal H_2_ production from water has been reported, sacrificial agents such as alcohols are necessary in these reports. Plasmonic photothermal pure water splitting is more promising but rarely reported. Future efforts are highly recommended to focus on this area.

#### 3.3.2 CO_2_ reduction with H_2_

**Fig. 6:**
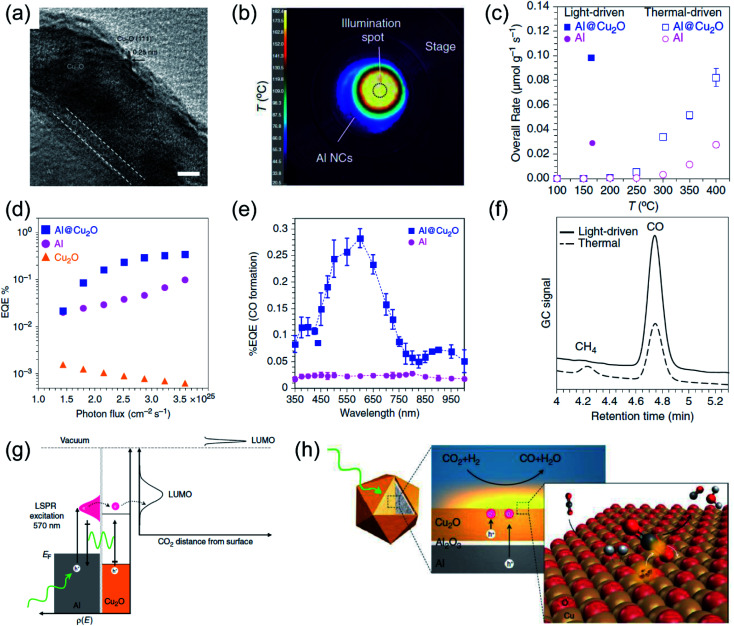
The plasmonic photothermal catalytic RWGS reaction over Al/Al_2_O_3_/Cu_2_O antenna-reactor nanoparticles. (a) A HRTEM image of Al/Al_2_O_3_/Cu_2_O. (b) The spatial temperature distribution on the surface of the catalyst under light irradiation. (c) A comparison of the overall rates of product formation under purely light-driven and thermal-driven conditions. (d) Apparent external quantum efficiency (EQE) plotted against photon flux. (e) Apparent external quantum efficiency (EQE) plotted against illumination wavelength. (f) Gas chromatograms of the chamber output during solar- and thermal-driven processes on Al@Cu_2_O. (g) The proposed mechanism of using Al@Cu_2_O for plasmon-mediated charge transfer and adsorbate activation processes. (h) A schematic illustration of hot electron-driven RWGS over Al@Cu_2_O plasmonic photothermal catalysts. (a–h) Reproduced from ref. 50 with permission from Springer Nature, Copyright 2017.

Halas and co-authors reported Al@Cu_2_O antenna-reactor plasmonic photothermal catalysts, which combined plasmonic light harvesting (Al) and active sites (Cu_2_O) to achieve an efficient plasmonic photothermal RWGS reaction.^50^ The Al@Cu_2_O catalysts were prepared by growing a Cu_2_O layer around an Al core. The Cu_2_O layer and Al core were separated by a self-limiting amorphous Al_2_O_3_ layer (2–4 nm in thickness) (Fig. 6a). During the activity measurements, a supercontinuum fiber laser was used as the illuminant without external heating. The surface temperatures of the catalysts under light irradiation were recorded by a thermal imaging camera, and a high surface temperature was detected over Al@Cu_2_O catalysts under irradiation by visible light at a light intensity of 10 W cm^−2^ (Fig. 6b). Through control experiments by comparing the activities under purely thermal-driven conditions and a photo-induced process, the contribution of plasmon-induced hot carriers in the photothermal RWGS reaction was differentiated from photothermal heating. Under irradiation by visible light at the maximum intensity (10 W cm^−2^) without additional energy input, a significantly higher overall reaction rate can be achieved compared with the reaction rate under purely thermal conditions, and even the surface temperature was significantly lower under visible light irradiation (Fig. 6c). Based on contrast experiment results, the authors indicated that the photothermal heat played a secondary role in the photocatalytic RWGS. In order to verify the participation of the photo-induced hot carriers on the surface reaction, several experiments under different conditions were conducted. The positive dependence of external quantum efficiencies (EQE) on incident photon flux, which is a distinct feature of a hot carrier-driven reaction, was observed (Fig. 6d). The contribution of hot carriers was also confirmed by wavelength-dependent activity measurement (Fig. 6e). Furthermore, with the assistance of photo-induced hot carriers, the reaction rate was enhanced significantly, and the selectivity for CO production was greatly improved in the light-driven process (Fig. 6f). Based on the experimental and theoretical investigations, the authors proposed that the hot carriers produced by the plasmonic Al were transferred to Cu_2_O through non-radiative plasmon decay in the Al core and near-field excitation of carriers in Cu_2_O (Fig. 6g). After that, abundant hot carriers were injected into the unoccupied states of CO_2_ adsorbed on the surface of Cu_2_O for C–O bond activation, which promoted the RWGS reaction (Fig. 6h). In this work, the role of photo-induced hot carriers in the reaction was systematically investigated, and a hot carrier-mediated reaction selectivity was observed. Through combining the contribution of photo-induced hot carriers and photothermal heat, a far more efficient catalytic activity is observed compared with the conventional thermocatalytic process, demonstrating the unique property of hot carriers for activating certain chemical bonds of the adsorbates towards more selective and efficient reaction pathways that cannot be accessed by the conventional thermocatalytic process.

**Fig. 7:**
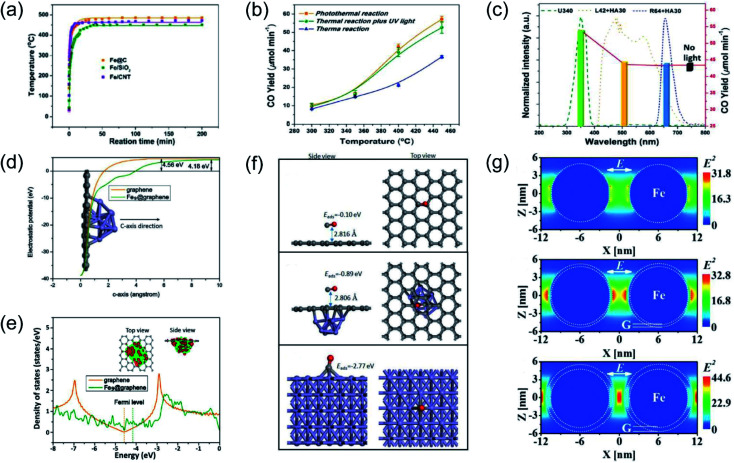
Plasmonic photothermal catalytic RWGS. (a) *In situ* monitoring of the surface temperature under light irradiation. (b) Comparison of CO generation rates for photo-induced thermal and conventional thermal reactions over an Fe@C catalyst. (c) CO production rates over Fe@C without or with light irradiation in different wavelength ranges at 450 °C. (d) The electrostatic potentials of graphene and Fe_9_@graphene. (e) Density of states (DOS) calculations involving graphene and Fe_9_@graphene. (f) CO adsorption configurations on graphene, Fe_9_@graphene, and Fe (110) surfaces. (g) The spatial distribution of the SPR-induced enhancement of the electric field over an Fe nanoparticle, Fe coated with a single layer of carbon, and Fe coated with multiple layers of carbon. (a–g) Reproduced from ref. 102 with permission from John Wiley and Sons, Copyright 2016.

Our group previously reported that photo-induced hot electrons over plasmonic Fe nanometals coated with a carbon layer (Fe@C) can effectively assist photothermal RWGS.^102^ Photoirradiation could induce significant heating through efficient photothermal conversion, since a high surface temperature (481 °C) on Fe@C was detected after light irradiation (Fig. 7a). After light irradiation for 2 h, a CO amount of 2196.17 μmol was obtained over the Fe@C catalyst. The authors proposed that apart from photo-induced heat, the hot carriers generated from Fe nanoparticles also participated in the surface reaction. A series of contrast experiments were conducted to reveal the difference between plasmonic photothermal catalysis (without additional thermal energy input) and conventional thermocatalysis (without light irradiation). Interestingly, the production rates for CO under the photo-induced conditions were obviously higher than those under thermal conditions at the same surface temperatures, suggesting the existence of another reaction mechanism other than photo-induced heat. Afterward, UV light was introduced into the reactor during the conventional thermocatalytic process, and a comparable activity to the photoinduced thermal condition was observed, indicating that UV light played a major role in enhancing the RWGS reaction over Fe@C catalysts (Fig. 7b). This result was further confirmed by contrast experiments under irradiation by different wavelengths (Fig. 7c). To investigate the underlying reaction mechanism, several types of theoretical calculation were conducted. The electrostatic calculation results showed that the work function of carbon atoms bonded to Fe_9_ was decreased by 0.38 eV after loading on graphene (Fig. 7d), which can be attributed to the charge transfer from Fe to graphene. As revealed by density of states (DOS) calculation, the electronic structure near the Fermi level was modified to a metallic state, resulting in a much easier electron transfer from Fe nanoparticles to reactants during the catalytic reaction (Fig. 7e). The adsorption energy of CO on different material surfaces was further investigated, and the results indicated that adsorption of CO on Fe can be modulated effectively after graphene coating, resulting in much easier desorption of CO (Fig. 7f). Electromagnetic-field simulation results revealed that the plasmon–photon coupling was amplified on the surface of the Fe nanoparticles. After coating a few layers of carbon over Fe, the electric field enhancement of Fe was further increased, facilitating the production of hot electrons for activating CO_2_ electronically (Fig. 7g). In this study, UV-light was captured by Fe nanoparticles to excite the LSPR effect, and the light-induced thermal effect can further promote the reaction. Through rational design of plasmonic photothermal catalysts, hot carrier activation, photothermal conversion and promoted desorption of CO were all integrated effectively in one catalyst, resulting in a highly efficient RWGS reaction.

Plasmonic Au was also utilized as a plasmonic photothermal catalyst for driving RWGS with high selectivity without addition thermal energy input. Jia and co-authors fabricated Au/CeO_2_ nanocomposites, and found that the reaction rate under photothermal conditions was much higher than under purely thermal conditions.^103^ Under visible light irradiation, a maximum surface temperature of 400 °C was detected, and the CO_2_ conversion reached 40%, which was approximately ten times higher than that in the thermal process. Contrast experiments under photothermal and purely thermal conditions revealed a decisive function of Au for high CO_2_ conversion in the photothermal process. Based on reaction kinetic investigations and the operando DRIFT spectra results, the authors concluded that the photo-induced hot electrons of Au can induce the dissociation of adsorbed H_2_, which favored the subsequent hydrogenation of CO_2_ to CO.

#### 3.3.3 CO_2_ reduction with CH_4_

Beyond the RWGS reaction, CO_2_ reduction with CH_4_ (dry reforming of methane, DRM), an industrially valuable but strongly endothermic reaction, can also be stimulated efficiently through plasmonic photothermal technology.

**Fig. 8:**
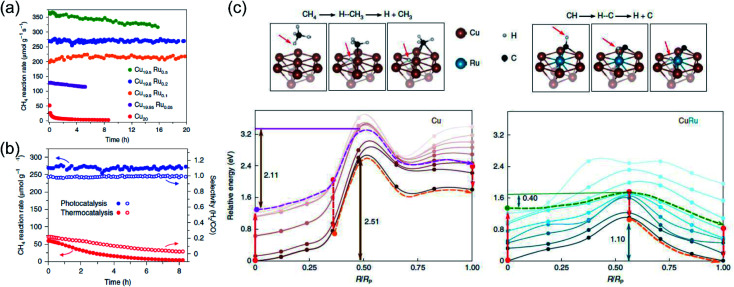
The plasmonic photothermal catalytic DRM reaction over Cu–Ru surface alloy antenna-reactor plasmonic photocatalysts. (a) Reaction activity and long-term stability data for Cu–Ru catalysts with different Ru ratios for plasmonic photothermal catalytic DRM. (b) A comparison of the activity, stability, and selectivity of Cu_19.8_Ru_0.2_ under 19.2 W cm^−2^ white-light illumination and for thermocatalysis at a reaction temperature of 1000 K. (c) Calculated ground- and excited-state energy curves for CH_4_ and CH dehydrogenation over Cu and Cu–Ru, respectively. (a–c) Reproduced from ref. 48 with permission from Springer Nature, Copyright 2020.

Very recently, Halas and co-authors designed a plasmonic single atomic site antenna-reactor consisting of a Cu nanoparticle “antenna” with single Ru active “reactor” sites dispersed on the Cu surface (Cu–Ru) for an efficient plasmonic photothermal DRM reaction.^48^ The Cu–Ru was synthesized through the co-precipitation method, and the catalytic activity towards photothermal DRM was measured in a flow-type reactor under atmospheric pressure. A supercontinuum laser was applied as the energy supply to drive the photothermal DRM reaction. The surface temperatures of the catalysts under illumination were recorded by a thermal camera. Under irradiation, a surface temperature of 1000 K was detected over Cu_19.8_Ru_0.2_. Cu_19.8_Ru_0.2_ demonstrated the optimal performance towards photothermal DRM among all the samples, and a turnover frequency of 34 mol H_2_ (mol Ru)^−1^ s^−1^ was achieved under irradiation by white light (19.2 W cm^−2^) without an external energy input (Fig. 8a). Through a comparison of activity and selectivity over Cu_19.8_Ru_0.2_ under photo-driven and thermal-driven conditions at the same surface temperature induced by illumination and external heating, a hot carrier-mediated DRM process was proposed (Fig. 8b). The participation of hot carriers in the surface reaction was further confirmed by investigating the reaction selectivity under different light intensities, and an increased selectivity with light intensity was observed, which was consistent with the hot carrier-mediated reaction mechanism. In a such process, a solar-to-fuel energy conversion efficiency of up to 15% was achieved under irradiation by white light with a strong light intensity of 16 W cm^−2^. Based on mechanistic investigations and theoretical calculations in the both ground- and excited-states, the authors indicated that the advanced plasmonic photothermal catalytic activity of Cu_19.8_Ru_0.2_ can be attributed to two aspects. Firstly, atomically dispersed Ru active sites on the surface of Cu facilitated CH_4_ dehydrogenation with a suppressed side reaction (such as RWGS) and coke formation. Secondly, C–H bond activation and H_2_ desorption on the Ru active sites were promoted by the photo-induced hot carriers, resulting in a substantial reduction of the energy barrier for CH_4_ activation and eventually facilitating the charge transfer onto the detaching H (Fig. 8c). Under intense white light irradiation, the plasmonic Cu could deliver the hot carriers to the surface Ru atoms, powering the surface reaction over Ru active sites. This study highlighted that light-energy might be a more effective form of energy input for driving DRM. By utilizing plasmonic photothermal catalysis, a combination of photo-induced hot carriers and photothermal heating can be achieved for driving tough but industrially valuable reactions.

## 4. Conclusions and outlook

The above studies conducted so far have demonstrated that plasmonic energy can serve as a promising and effective stimulus for converting solar-energy into chemical energy. The full solar-spectrum, including ultraviolet, visible, and even infrared light, can be effectively harnessed by plasmonic catalysts for driving various kinds of reactions, such as H_2_O splitting, CO_2_ reduction, and artificial photosynthesis. Depending on the different decay pathways of plasmonic energy, the main driving force for initiating the surface reaction can be disparate, classifying the reaction process into plasmonic photocatalysis, photothermal catalysis and plasmonic photothermal catalysis. During plasmonic photocatalysis where the hot carriers play dominant roles, the solar-to-fuel conversion efficiencies are unsatisfactory. Compared with plasmonic photocatalysis, photothermal catalysis demonstrates better efficiency for solar-to-fuel conversion due to the participation of photothermal heat in the surface reactions, but the activation of adsorbates by hot carriers is negligible. Furthermore, with the assistance of both the hot carrier-induced adsorbate activation and photothermal heating, plasmonic photothermal catalysis enables the initiation of thermodynamically unfavorable reactions efficiently and selectively compared with both conventional photocatalytic and thermocatalytic processes, resulting in a highly boosted STF conversion efficiency. The utilization of plasmonic photothermal technology presents several advantages. First, the most abundant and renewable solar-energy is adopted in the chemical reaction as the driving force, which can effectively reduce the consumption of non-renewable fossil fuels. Second, the reaction pathways can be modified by injecting hot carriers into specific chemical bonds, thus increasing the reaction selectivity. Third, compared with the thermocatalytic process, the milder reaction conditions of the photothermal catalytic process can lead to significant advantages, such as prolonged stability, enhanced safety, and a wider choice of catalysts. Although there have been significant developments in plasmonic catalysis in the past few years, both the STF and AQE efficiency are still rather moderate, due to the limited light-capturing ability and disordered charge transfer process. In order to further promote the efficiency, it is highly desirable to develop plasmonic nanostructures with excellent light response from UV to infrared light, numerous active catalytic sites, and prolonged lifetimes of hot carriers by the modification of plasmonic nanostructures, such as modulating the size and shape of plasmonic metals, and the construction of complex (potentially multi-metallic) plasmonic nanostructures.

First, the charge transfer behavior of plasmonic nanostructures is an essential factor for determining the overall catalytic activity and selectivity. Such a phenomenon is attributed to the specific injection of hot carriers from photocatalysts to certain chemical bonds of the adsorbates, resulting in a new mechanistic pathway through the selective initiation of individual elementary steps of chemical reactions. Current investigations suggest that the energy of hot carriers could be modulated by tailoring the optical properties of plasmonic nanostructures (such as SPR band position and intensity) in order to match the specific electronic states and/or vibrational modes of adsorbates, ultimately controlling the reaction selectivity precisely. Toward this end, significant advances in the design and fabrication of plasmonic nanostructures with optimal geometry and composition will be highly desirable to realize a controllable charge transfer process. For instance, combining the plasmonic metal with another chemically reactive metal is considered to be promising for constructing highly efficient plasmonic photothermal catalysts. The optical property of the catalysts is determined by plasmonic metals, and the interaction between adsorbates and reactive metals enables the tuning of the electronic structure of the adsorbates. By matching these optical and electronic properties, the charge transfer behavior over plasmonic metals can be regulated, inducing a preferential charge carrier injection to particular orbital of adsorbates. In addition, great efforts should also be devoted to the development of predictive theories and a basic understanding of the molecular behavior on the surface of plasmonic metals, which will be helpful for us in designing plasmonic photothermal catalysts with optimal nanostructures and compositions.

Second, apart from hot carrier excitation and adsorbate activation, photothermal conversion is also a critical parameter which influences the performance of plasmonic photothermal catalysts. The optical characteristics (such as position of SPR absorption peak and light absorption capacity) of plasmonic nanostructures are the fundamentals to determine the photon energy that can be utilized for participating in the reaction. As for the aspect of photothermal conversion, transforming solar-energy across the entire solar-spectrum, especially low-energy photons in the visible and infrared regions, into heat should be more favorable. One potential avenue for achieving this is to synthesize complex (potentially multi-metallic) plasmonic nanostructures to create more oscillation modes and hotspots, resulting in a full solar-spectrum response. In addition, modification of the nanostructures to improve antireflection may result in enhanced sunlight-harvesting ability, improving the photothermal conversion.

Third, plasmonic photothermal catalysis is a newly developed technology in the broad area of catalysis. Basic understandings of the reaction mechanism, such as hot carrier generation and reactant activation on the plasmonic nanostructures, remain at the preliminary stage owing to the complicated reaction pathways and limited surface characterization techniques. Advanced mechanistic investigations, such as excited-state theoretical calculation and *in situ* transient surface analysis are expected to provide some convincing evidence for unravelling the interaction process between plasmonic nanostructures and reactants. In addition, in the plasmonic photothermal catalysis system, photothermal heat and hot carrier transfer are likely to act in concert, and the contribution of each factor remains to be clarified qualitatively and quantitively. In order to investigate the underlying thermodynamics and kinetics of plasmonic photothermal catalytic reactions, more technologies are required for not only accurately measuring the surface temperature of catalysts, but also for identifying the transient species generated during the reaction.

Finally, in addition to plasmonic photothermal catalysis that utilizes the full solar spectrum efficiently, it is important to explore other strategies that could combine both light and thermal features of solar energy for highly efficient solar-to-energy conversion. The recently developed solar thermal electrochemical process (STEP) provides an alternative and effective strategy for solar-to-chemical conversion, especially in CO_2_ reduction.^104^ In STEP, solar light is concentrated and separated into ultraviolet, visible, and infrared light, in which the ultraviolet-visible portion is utilized to drive a photovoltaic (PV) device to generate electricity for electrolysis, and the infrared portion is utilized to heat an electrolysis cell.^105,106^ As a result, the required electrical potential can be lowered enormously in the electrolysis process when the reaction temperature rises drastically under infrared light irradiation.^107^ Several electrolysis reactions which were originally initiated at room temperature, such as CO_2_ reduction to produce CO or CH_4_, can be promoted at high temperature *via* the solar thermal electrochemical process.^108–110^ STEP provides an alternative strategy for solar-energy conversion, with full utilization efficiency of solar light. At present, several important factors, such as the electrolysis temperature and electrolyte composition, should be extensively investigated to achieve higher product selectivity and conversion efficiency. In addition, it is highly desirable to develop more stable electrode materials to maintain long-term operation under the harsh reaction conditions of STEP.

## Author contributions

H. S. and J. Y. conceived the overall idea of the perspective. S. L., X. R., H. L. wrote the initial manuscript. S. L., H. S. and J. Y. revised and edited the manuscript.

## Conflicts of interest

There are no conflicts to declare.
